# The effect of action observation combined with high-definition transcranial direct current stimulation on motor performance in healthy adults: A randomized controlled trial

**DOI:** 10.3389/fnhum.2023.1126510

**Published:** 2023-03-01

**Authors:** Gidon Schwell, Zvi Kozol, David Tarshansky, Moshe Einat, Silvi Frenkel-Toledo

**Affiliations:** ^1^Department of Physical Therapy, School of Health Sciences, Ariel University, Ariel, Israel; ^2^Department of Electrical and Electronic Engineering, Ariel University, Ariel, Israel; ^3^Department of Neurological Rehabilitation, Loewenstein Rehabilitation Medical Center, Ra’anana, Israel

**Keywords:** action observation, tDCS, motor performance, mirror neuron system, reaching sequence

## Abstract

Action observation (AO) can improve motor performance in humans, probably *via* the human mirror neuron system. In addition, there is some evidence that transcranial direct current stimulation (tDCS) can improve motor performance. However, it is yet to be determined whether AO combined with tDCS has an enhanced effect on motor performance. We investigated the effect of AO combined with high-definition tDCS (HD-tDCS) targeting the inferior parietal lobe (IPL) and inferior frontal gyrus (IFG), the main aggregates of the human mirror neuron system, on motor performance in healthy adults and compared the immediate vs. 24-h retention test effects (anodal electrodes were placed over these regions of interest). Sixty participants were randomly divided into three groups that received one of the following single-session interventions: (1) observation of a video clip that presented reaching movement sequences toward five lighted units + active HD-tDCS stimulation (AO + active HD-tDCS group); (2) observation of a video clip that presented the same reaching movement sequences + sham HD-tDCS stimulation (AO + sham HD-tDCS group); and (3) observation of a video clip that presented neutral movie while receiving sham stimulation (NM + sham HD-tDCS group). Subjects’ reaching performance was tested before and immediately after each intervention and following 24 h. Subjects performed reaching movements toward units that were activated in the same order as the observed sequence during pretest, posttest, and retest. Occasionally, the sequence order was changed by beginning the sequence unexpectedly with a different activated unit. Outcome measures included mean Reaching Time and difference between the Reaching Time of the unexpected and expected reaching movements (Delta). In the posttest and retest, Reaching Time and Delta improved in the AO + sham HD-tDCS group compared to the NM + HD-sham tDCS group. In addition, at posttest, Delta improved in the AO + active HD-tDCS group compared to the NM + sham HD-tDCS group. It appears that combining a montage of active HD-tDCS, which targets the IPL and IFG, with AO interferes with the positive effects of AO alone on the performance of reaching movement sequences.

## 1. Introduction

It is possible to acquire novel motor skills or to improve motor performance by observing the actions of others (Mattar and Gribble, 2005; Osman et al., 2005; Porro et al., 2007; Lee et al., 2013; Kim et al., 2017). Action observation (AO) has been found to improve a variety of motor tasks, such as serial reaction time tests (Osman et al., 2005) and force production (Porro et al., 2007), among healthy subjects and populations with neurological (Pelosin et al., 2010, 2013; Buccino et al., 2011; Lee et al., 2013; Park and Hwangbo, 2015) and orthopedic conditions (Paravlic, 2022) (however, see also Sarasso et al., 2015; Gatti et al., 2019; Borges et al., 2022).

The effects of AO on motor performance are thought to be mediated *via* the human mirror neuron system (hMNS) (Rizzolatti and Craighero, 2004; Buccino, 2014; Kemmerer, 2021; Kim and Lee, 2021). Mirror neurons are specific neurons that are activated while performing motor actions and observing those actions (Rizzolatti et al., 1996). They were first found in macaque monkeys in the ventral premotor cortex (F5) and around the anterior intra-parietal sulcus of the macaque (di Pellegrino et al., 1992; Fogassi et al., 2005). The hMNS is thought to reside in a network comprised of the inferior frontal gyrus (IFG), the inferior parietal lobule (IPL) and additional cortical regions such as the ventral premotor cortex and superior parietal lobule (e.g., Grèzes et al., 2003; Filimon et al., 2007; Gazzola et al., 2007; Gazzola and Keysers, 2009; Caspers et al., 2010; Molenberghs et al., 2012; Kemmerer, 2021).

In addition to AO, another means that can improve motor performance in healthy adults (Patel et al., 2019; Halakoo et al., 2020; Wang et al., 2021) and populations with neurological conditions (Broeder et al., 2015; Ammann et al., 2016; Kang et al., 2016; Sánchez-Kuhn et al., 2017) is transcranial direct current stimulation (tDCS). It should be noted, however, that some studies did not find tDCS-related positive effects on motor performance (for reviews see Elsner et al., 2017; Lefaucheur et al., 2017). This is a safe stimulation method that delivers weak direct currents (usually 0.5–2 mA) *via* surface electrodes placed on the skull. It alters spontaneous brain activity and excitability by subthreshold modulation of neuronal membranes in a polarity-dependent manner (Roche et al., 2015; Stagg et al., 2018). It is assumed that anodal stimulation enhances cortical excitability, while cathodal stimulation reduces it (Nitsche and Paulus, 2000; Stagg et al., 2018); however, the effects of tDCS are much more complex. For instance, a non-linear dose–response relationship in neurophysiological (Nitsche and Paulus, 2000; Batsikadze et al., 2013; Moliadze et al., 2015; Ammann et al., 2016; Strube et al., 2016) and behavioral measures (Ehrhardt et al., 2021; Lerner et al., 2021) was found. To avoid the assumption that anodal stimulation necessarily reflects excitatory stimulation, we have described the term anodal tDCS in this paper as active tDCS, meaning that an anodal electrode was placed over the region of interest (ROI).

Although the separate effects of AO and tDCS on motor performance have been extensively investigated (Wang et al., 2021), to the best of our knowledge, the combination of tDCS and AO has been implicated in only a few behavioral studies (Wade and Hammond, 2015; Apšvalka et al., 2018) and neurophysiological studies (Feurra et al., 2011; Enticott et al., 2012; Qi et al., 2019). From a neurophysiological viewpoint, reduced motor evoked potentials (MEPs) have been found following AO combined with 10 min of 1 mA conventional tDCS (which uses large pad electrodes), with a cathodal electrode placed over M1 (Qi et al., 2019), as well as during AO conducted immediately following 10 min of 1.5 mA conventional tDCS with an anodal electrode placed over the parietal cortex (Feurra et al., 2011). Conventional 2 mA tDCS, for 20 min, with an anodal or cathodal electrode placed over the IFG, but not IPL, reduced MEP amplitude (Enticott et al., 2012). From a motor behavior viewpoint, 20 min of conventional 1 mA tDCS with an anodal electrode placed over M1 during AO did not significantly affect keypress sequence performance (Apšvalka et al., 2018). In contrast, 15 min of 1 mA conventional tDCS with an anodal electrode placed over the left premotor cortex improved reaction time without losing accuracy of a serial response time task in the posttest compared to the sham stimulation group (Wade and Hammond, 2015).

As IPL and IFG are considered to be the main aggregates of the hMNS (e.g., Grèzes et al., 2003; Filimon et al., 2007; Gazzola et al., 2007; Gazzola and Keysers, 2009; Caspers et al., 2010; Molenberghs et al., 2012; Kemmerer, 2021), this study is the first attempt to determine the effect of AO combined with tDCS that simultaneously targets IPL and IFG on motor performance among healthy adults, relating to the immediate vs. retention test effects. Most of the tDCS research, including studies dealing with the effect of AO combined with tDCS, used conventional large pad tDCS. Conventional tDCS delivers current to diverse brain regions, rather than only to the targeted ROI. Improved spatial focality of tDCS can be achieved using high-definition (HD) tDCS (Datta et al., 2009, Datta et al., 2013; Caparelli-Daquer et al., 2012; Kuo et al., 2013). Compared to conventional large pad tDCS, HD-tDCS demonstrated a peak induced electric field magnitude at the sulcus and adjacent gyri directly below the active electrode (Datta et al., 2009). Therefore, using HD-tDCS, which allows more nuanced control of current flow to both the IFG and IPL during AO, may be more beneficial for determining the effects of stimulating these ROIs on motor performance (although it does not eliminate the current spatial distribution). We compared the effects of AO *via* a video clip combined with active HD-tDCS that targets the IPL and IFG, AO *via* a video clip combined with sham HD-tDCS, and observation of a neutral movie (NM) clip combined with sham HD-tDCS on reaching movement sequences. We hypothesized that (1) AO combined with active HD-tDCS would be more effective in improving motor performance than AO combined with sham HD-tDCS and observing NM combined with sham HD-tDCS and (2) AO combined with sham HD-tDCS would be more effective in improving motor performance than NM combined with sham HD-tDCS.

## 2. Materials and methods

### 2.1. Study design

This was a single-blind, parallel, randomized, sham-controlled study. Data were collected in a brain and motor behavior laboratory at Ariel University, Israel. Subjects were randomly assigned with a 1:1:1 ratio, using a random number generator in WINPEPI, to one of three groups: (1) AO + active HD-tDCS (AO + active HD-tDCS group); (2) AO + sham HD-tDCS (AO + sham HD-tDCS group); and (3) Observation of NM + sham HD-tDCS (NM + sham HD-tDCS group). All participants were blinded to group allocation. The stimulator monitor was hidden from the participants, and the sham stimulation increased and decreased in a ramp-like fashion to ensure blinding of participants (see HD-tDCS section).

### 2.2. Participants

Sixty participants participated in the study (30 women, aged 24 ± 3 years). Inclusion criteria included right-hand dominance and normal health status according to self-report. Exclusion criteria included musculoskeletal or neurological deficits affecting task performance (proper reaching performance in a sitting position). Participants signed an informed consent form prior to participating in the study. All procedures were approved by the Ariel University Institutional Ethical Board (approval number: AU-HEA-SFT-20210721) and were performed in accordance with relevant guidelines and regulations. Participants were paid $20 for their participation.

### 2.3. Motor task

In all participants, the left arm was tested. The non-dominant arm was tested to challenge the motor performance of healthy participants, aiming to allow more room for improvement in the motor performance. This approach is acceptable in motor learning studies (e.g., Korman et al., 2007; Friedman and Korman, 2016). The participants performed a sequential reaching task. A detailed description of the task and the apparatus is provided in a previous study (Frenkel-Toledo et al., 2020). Subjects from each group participated in a session that included familiarization with the motor task, pretest, single session intervention (AO + active HD-tDCS/AO + sham HD-tDCS/NM + sham HD-tDCS), posttest, and retention test after 24 h.

**FIGURE 1:**
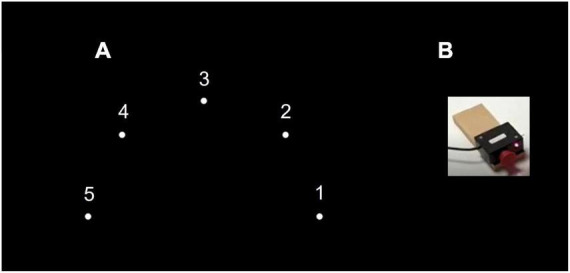
Experimental setup. **(A)** General setup of the motor task. **(B)** Push-button switch and a red light-emitting diode (LED), were attached to the tabletop in a 38-cm radius half circle, numbered right to left from 1 to 5.

Apparatus used in the tests: The custom-made testing device was set up on a table with a smooth tabletop of 105 cm × 80 cm × 80 cm. Five switch-led units of 5 cm × 8 cm × 5 cm, each composed of a large push-button switch and a red light-emitting diode (LED), were connected to the tabletop in a 38-cm radius half circle, numbered right to left from 1 to 5 (see Figure 1).

The system was operated by a desktop computer interfaced with a data acquisition card of LabVIEW software. The algorithm enables parameter selection of the LED activation (illumination) sequence, duration of reaching movement (RM), delay between RMs, and number of RM repetitions. Participant reached toward the activated unit LED and press the push-button switch. Pushing the switch of an activated unit deactivated it, and the response time between the activated and deactivated LED was recorded. The testing position of the participants was sitting on a chair with solid back support in front of the table, hips and knees flexed 90°. The starting position included placing the left fist of the participants on the edge of the table in front of their chest (parallel to switch three) so that they could reach and touch switch three with their third metacarpal.

**FIGURE 2:**
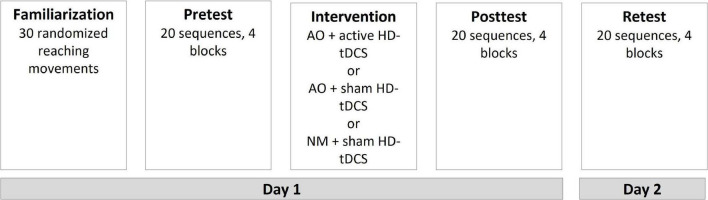
Experimental procedure. AO, action observation; HD-tDCS, high-definition transcranial stimulation; NM, neutral movies.

The familiarization practice of the participants consisted of 30 randomized RMs toward the activated unit, touching the unit-related switch as fast as possible, and returning the hand to the starting position until the next unit was activated. During each pretest, posttest, and retest, the participant was asked to perform RMs toward the units as fast and accurately as possible (they were not told that the task consisted of a reaching sequence). The units were activated in the sequence 1, 4, 3, 5, 4, 2 and with an activation duration and delay of 1 s. If the participant did not reach the activated unit within 1 s or if an incorrect unit was pressed, the trial was considered a “fail” and was not included in the averaged response time. During pretest, posttest, and retest, the participants performed 20 sequences (i.e., 120 RMs); five sequences constituted each block. The participants rested for 30 s after each block. In the fifth, third, fourth, and second sequences of the first, second, third, and fourth blocks, respectively, the sequence order of the test was changed unexpectedly and began with unit 5 instead of unit 1; that is, 5, 4, 3, 5, 4, 2 instead of 1, 4, 3, 5, 4, 2. Response times toward all units were recorded. The averaged response times of the RMs toward all the targets and toward unit 5 during the regular sequence and the unexpected sequence (the latter relates to RM toward the first five in the unexpected sequence 5, 4, 3, 5, 4, 2) were recorded (see Figure 2).

Outcome measures: Reaching Time (milliseconds) was computed by averaging the response time of all the RMs during the sequences, and Delta (milliseconds) was computed by subtracting the response time of the expected RMs from the unexpected RMs toward unit 5. The time limit for each RM is 1 s. As for each pretest, posttest, and retest Delta was averaged across four trials only (one trial per block), specifically for Delta, the value 1,001 ms was given for a RM toward the unexpected unit 5 that was not accomplished within 1 s (for a similar approach, see Bogard et al., 2009; Fritz et al., 2009; Frenkel-Toledo et al., 2020). Improved motor performance was indicated by a shorter Reaching Time and a larger positive time difference between the unexpected and expected RMs toward unit 5 (higher Delta).

### 2.4. Action observation

The participants were instructed to observe the video clip and avoid moving during the observation. In the AO + active HD-tDCS and AO + sham HD-tDCS groups, the observed sequence (7 s each) in the video clip consisted of six RMs toward the units in the order of 1, 4, 3, 5, 4, 2 (averaged response time of the observed RM: 126.83 ± 37 ms). The units were activated in the same order as in the pretest, posttest, and retest. The observed RM sequence was executed by a young healthy male (27 years), performing the above sequence with his non-dominant left hand on the same device used by the participants. The participants observed 120 sequences (720 RMs) from an egocentric viewpoint, as it was found to be more effective than an allocentric viewpoint with respect to behavioral imitation tasks (Watanabe and Higuchi, 2016) and neurophysiological measures (Angelini et al., 2018), with 10 s rest periods after every 20 sequences. In the NM + sham HD-tDCS, participants observed NM that consisted of nature views without any human or animal movements, with 10 s rest periods of a blank screen at the same rest time points as during the AO video. The intervention lasted 15 min.

### 2.5. High-definition-tDCS

**FIGURE 3:**
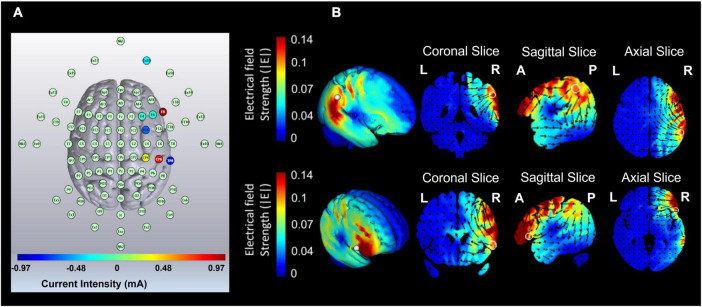
Current flow modeling during 1 mA high-definition transcranial direct current stimulation (HD-tDCS) using the HD-Target software (Soterix Medical, New York, NY). **(A)** High-definition transcranial direct current stimulation (HD-tDCS) montage for maximal focal stimulation of the right inferior parietal lobe (IPL) and the right inferior frontal gyrus (IFG). **(B)** Current flow modeling during 1 mA high-definition transcranial direct current stimulation (HD-tDCS) using HD-Target software (Soterix Medical, New York, NY). Current-flow models of the right IPL and the right IFG are shown on 2D and 3D reconstructions of the cortical surface. Skin, skull, and cerebrospinal fluid (CSF) masks are suppressed to reveal the underlying gray matter mask. A head model derived from the MNI 152 dataset was used. L, left; R, right; A, anterior; P, posterior.

The stimulation was administered non-invasively using an M × N 9-channel HD transcranial electrical current stimulator from Soterix Medical (New York, NY). Eight sintered Ag/AgCl electrodes were attached to plastic holders, filled with conductive gel, and embedded in a HD cap, according to the extended 10–20 method of electrode placement. HD-Targets brain modeling software (Soterix Medical, New York, NY) was used to determine the tDCS montage for maximal focal stimulation of the right IPL and right IFG (Figure 3). We administered a single session of 15 min of active stimulation at 1 mA (Wade and Hammond, 2015) targeting the IPL and the IFG by positioning electrodes at the following sites with the following intensities: F4 (0.48 mA), F8 (−0.08 mA), FC4 (−0.29 mA), CP4 (0.11 mA), F6 (−0.1 mA), CP6 (0.41 mA), TP8 (−0.36 mA), and Ex18 (−0.17 mA). In the stimulation group, the current gradually increased over the course of the first 30 s and gradually decreased over the last 30 s. In the sham groups, once the current reached 1 mA over the first 30 s, it was ramped back down over 30 s. In the last minute of the simulation, an identical ramp up and ramp down was administered (for a similar approach, see Ljubisavljevic et al., 2016; Charvet et al., 2018; Greeley et al., 2020; Lerner et al., 2021). Participants were asked to report any adverse effects and to rank their discomfort from 1 to 10 following two min of stimulation.

### 2.6. Statistical analysis

Age and sex were compared between groups (AO + active HD-tDCS; AO + sham HD-tDCS; NM + sham HD-tDCS) using One-Way ANOVA and chi-square tests, respectively. The differences between groups with respect to each of the main outcomes (Reaching Time, Delta) in the pretest were investigated using one-way ANOVA with Bonferroni correction for multiple comparisons. The effects of intervention and time on the main outcomes were investigated using mixed ANOVA with time (pretest, posttest, and retest) as a within-subject factor and group (AO + active HD-tDCS; AO + sham HD-tDCS; NM + sham HD-tDCS) as the between-subject factor with Bonferroni correction for multiple comparisons. Greenhouse–Geisser Epsilon (G-GE) was used to correct the degrees of freedom when Mauchly’s test of sphericity was significant. The differences between groups with respect to the frequency of adverse effects were investigated using a chi-squared test. The differences between groups with respect to discomfort from adverse effects were investigated using the Kruskal–Wallis test with Bonferroni correction for multiple comparisons (for a similar approach, see Swissa et al., 2022). Outcome measures were normally distributed. All tests were performed using SPSS (version 25.0) with initial significance levels of *p* < 0.05.

## 3. Results

**FIGURE 4:**
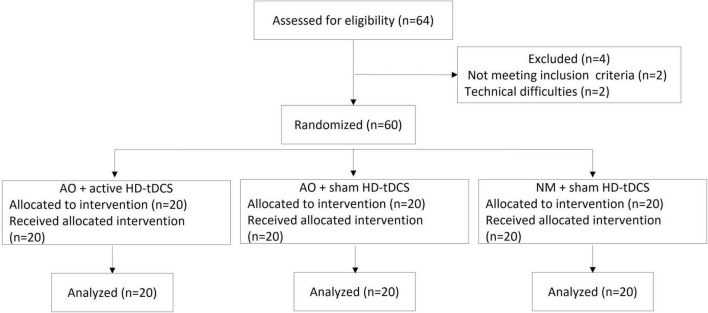
Trial flowchart. AO, action observation; HD-tDCS, high-definition transcranial direct current stimulation; NM, neutral movie.

The flowchart illustrating the process of the study is shown in Figure 4. Sixty-four participants were recruited for the study. Of those, two did not meet the inclusion criteria and two were excluded due to technical difficulties. No statistical differences were found between the groups in age (AO + active HD-tDCS: 23.5 ± 1.91 years; AO + sham HD-tDCS: 23.7 ± 2.86 years; NM + sham HD-tDCS: 24.6 ± 2.76 years) or gender (10 males in each group). Mean values and standard deviations of Reaching Time (milliseconds) and Delta (milliseconds) by group are shown in Table 1. No statistical difference was found between the groups in Reaching Time or Delta at the pretest (*p* > 0.38 for all).

**TABLE 1:** Mean values and standard deviations of measures reaching time and delta.

	AO + HD-tDCS (*n* = 20)			AO + sham HD-tDCS (*n* = 20)			NM + sham HD-tDCS (*n* = 20)		
	**Pretest**	**Posttest**	**Retest**	**Pretest**	**Posttest**	**Retest**	**Pretest**	**Posttest**	**Retest**
Mean reaching time (ms)	572.10 ± 88.57	523.32 ± 104.31	507.71 ± 115.18	544.49 ± 70.94	482.84 ± 74.53	453.14 ± 92.63	577.06 ± 62.09	562.65 ± 63.65	539.43 ± 63.16
Mean delta (ms)	−76.49 ± 59.51	93.35 ± 150.71	27.49 ± 127.30	−72.24 ± 97.86	114.18 ± 175.68	99.75 ± 155.94	−57.31 ± 55.96	−30.27 ± 116.13	−45.97 ± 80.74

ms, milliseconds; AO, action observation; HD-tDCS, high-definition transcranial direct stimulation; NM, neutral movie.

**FIGURE 5:**
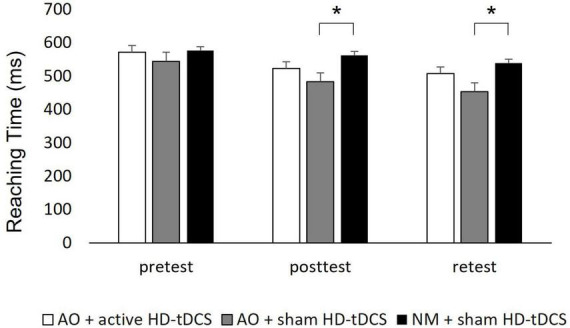
Mean of reaching time of each group displayed by time. Ms, millisecond; AO, action observation; HD-tDCS, high-definition transcranial direct stimulation. Error bars show the standard deviation. Asterisks denote a significant difference.

Reaching time: A main effect of Time [*F*_(2,114)_ = 55.142; *p* < 0.001; partial η2 = 0.49; observed power = 1.00] showed that across the groups, Reaching Time was significantly shorter at posttest (522.94 ± 87.62 ms) and retest (500.09 ± 98.04 ms) compared to the pretest (564.55 ± 74.82 ms; pBonferroni < 0.001 for all), and shorter at retest compared to posttest (pBonferroni < 0.001). A significant interaction of Group X Time [*F*_(4,114)_ = 3.921; *p* = 0.005; partial η2 = 0.12; observed power = 0.89] showed that the Reaching Rime differed significantly between groups in posttest [F_(2,59)_ = 4.663, *p* = 0.013] and in retest [*F*_(2,59)_ = 4.423, *p* = 0.016]. In posttest, Reaching Time was significantly shorter in the AO + sham HD-tDCS group (482.84 ± 74.53 ms) compared to the NM + sham HD-tDCS group (562.65 ± 63.65 ms; pBonferroni = 0.01). Similarly, in retest, Reaching Time was significantly shorter in the AO + sham HD-tDCS group (453.14 ± 92.63 ms) compared to the NM + sham HD-tDCS group (539.43 ± 63.16 ms; pBonferroni = 0.014) (see Figure 5 and Supplementary Figure 1). A main effect of Group [*F*_(2,57)_ = 3.583; *p* = 0.034; partial η2 = 0.11; observed power = 0.64] showed that across the time points, Reaching Time was significantly shorter at the AO + sham HD-tDCS group (493.49 ± 87.43 ms) compared to the NM + sham HD-tDCS group (559.71 ± 63.84 ms; pBonferroni = 0.031). In addition, in AO + active HD-tDCS group and AO + sham HD-tDCS group, response time decreased significantly in posttest (523.32 ± 104.31 ms and 482.84 ± 74.53 ms, respectively) and retest (507.71 ± 115.18 ms and 453.14 ± 92.63 ms, respectively) compared to the pretest (572.10 ± 88.57 ms and 544.49 ± 70.94 ms, respectively; pBonferroni < 0.001, for all). However, in NM + sham HD-tDCS group, Reaching Time decreased significantly in retest (539.43 ± 63.16 ms) compared to the posttest (562.65 ± 63.65 ms; pBonferonni = 0.005) and pretest (577.06 ± 62.09 ms; pBonferroni < 0.001). No other significant effects were observed.

**FIGURE 6:**
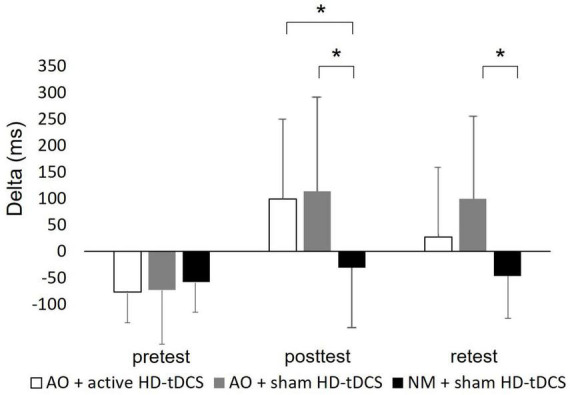
Mean of delta of each group displayed by time. ms, milliseconds; AO, action observation; HD-tDCS, high-definition transcranial direct stimulation. Error bars show the standard deviation. Asterisks denote a significant difference.

Delta: A main effect of Time [*F*_(2,114)_ = 29.467; *p* < 0.001; partial η2 = 0.34; observed power = 1.00] showed that across the groups, Delta was larger (positive) at the posttest (61.09 ± 160.87 ms) and retest (27.09 ± 136.92 ms, pBonferroni < 0.001 for all) compared to pretest (average delta: −68.68 ± 72.81 ms), and larger at posttest compared to retest (pBonferroni = 0.003). The significant interaction of Group X Time [*F*_(4,114)_ = 5.612; *p* < 0.001; partial η2 = 0.17; observed power = 0.95] showed that Delta differed significantly between groups in posttest [*F*_(2,59)_ = 5.65, *p* = 0.06] and at retest [*F*_(2,59)_ = 6.77, *p* = 0.02]. In posttest, Delta was significantly larger in the AO + active HD-tDCS group (99.35 ± 150.71 ms, pBonferroni = 0.024) and the AO + sham HD-tDCS group (114.18 ± 175.68 ms, pBonferroni = 0.01) compared to the NM + sham HD-tDCS group (−30.27 ± 116.13 ms). In retest, Delta was significantly larger in the AO + sham HD-tDCS group (99.74 ± 155.94 ms, pBonferroni = 0.002) compared to the NM + sham HD-tDCS group (−45.97 ± 80.73 ms) (see Figure 6 and Supplementary Figure 2). A main effect of Group [*F*_(2,57)_ = 5.238; *p* = 0.008; partial η2 = 0.16; observed power = 0.81] showed that across the time points, Reaching Time was significantly larger at the AO + sham HD-tDCS group (47.23 ± 167.77 ms) compared to the NM + sham HD-tDCS group (−44.52 ± 87.04 ms; pBonferroni = 0.031). In addition, only in AO + active HD-tDCS group and AO + sham HD-tDCS group, Delta differed between time points such that Delta increased (larger positive time) significantly in posttest (99.35 ± 150.71 ms and 114.18 ± 175.68 ms, respectively) and retest (27.49 ± 127.30 ms and 99.74 ± 155.94 ms, respectively) compared to the pretest (−76.49 ± 59.51 ms and −72.24 ± 97.86 ms, respectively; pBonferroni < 0.004, for all). Also, only in AO + active HD-tDCS group, Delta decreased significantly in retest compared to the posttest (pBonferonni = 0.009). No other significant effects were observed.

Adverse effects: The stimulation was well tolerated by the participants. The frequency and discomfort of adverse effects by group are displayed in Table 2. Frequency of adverse effects (tingling, burning, itching, and headache) did not differ between the groups. Strength of the discomfort from the adverse effects essentially did not differ between groups except for tingling (*p* = 0.001) such that it was significantly higher in the AO + HD-tDCS group (median: 3, interquartile range: 0–0) compared to the AO + sham HD-tDCS group (median: 0, interquartile range: 0–0; *p* = 0.016) and the NM + sham HD-tDCS group (median: 0, interquartile range: 0–0; *p* < 0.001).

**TABLE 2:** Frequency and discomfort of adverse effects.

Adverse effect	AO + HD-tDCS group (*n* = 20)		AO + sham HD-tDCS group (*n* = 20)		NM + sham HD-tDCS group (*n* = 20)	
	**Frequency**	**Discomfort**	**Frequency**	**Discomfort**	**Frequency**	**Discomfort**
Tingling	15 (75%)	3 (0–0)	7 (35%)	0 (0–0)	4 (20%)	0 (0–0)
Burning	3 (15%)	0 (0–0)	2 (10%)	0 (0–0)	0 (0%)	0 (0–0)
Itching	1 (5%)	0 (0–0)	5 (25%)	0 (0–0)	6 (30%)	0 (0–0)
Headache	0 (0%)	0 (0–0)	0 (0%)	0 (0–0)	1 (5%)	0 (0–0)

Median values and interquartile ranges of discomfort are presented. AO, action observation; HD-tDCS, high-definition transcranial direct stimulation; NM, neutral movie.

## 4. Discussion

The goal of the current study was to elucidate the immediate and 24-h retention test effects of AO combined with active HD-tDCS, targeting the IFG and IPL, on subsequent motor performance in healthy adults. In the posttest and retest, Reaching Time and Delta of the RM sequence improved in the AO + sham HD-tDCS group (but not in the AO + active HD-tDCS group) compared to the NM + sham HD-tDCS group. In addition, in the posttest, Delta improved in the AO + active HD-tDCS group compared to the NM + sham HD-tDCS group.

Our finding that Reaching Time and Delta of the RM sequence improved following AO with sham HD-tDCS compared to observation of the neutral movie with sham HD-tDCS is in line with our second hypothesis and previous evidence regarding immediate (Osman et al., 2005; Di Rienzo et al., 2019; Frenkel-Toledo et al., 2020) and off-line gains of observational learning (Porro et al., 2007; Trempe et al., 2011; Hesseg et al., 2016; Kim et al., 2017; Frenkel-Toledo et al., 2020) in healthy adults. The immediate and retention improvements can be related to an initial fast learning phase during the session and a slow, across-session phase due to consolidation, respectively (Karni et al., 1998; Robertson et al., 2005).

As opposed to our first hypothesis, AO, together with active HD-tDCS, in which anodal electrodes were placed over the IPL and IFG, did not improve Reaching Time compared to AO combined with sham HD-tDCS and observation of NM with sham HD-tDCS. Here, in contrast to previous studies that investigated the combined effects of AO and tDCS on motor performance (Wade and Hammond, 2015; Apšvalka et al., 2018), we placed electrodes over the IPL and IFG to specifically target the main aggregates of the hMNS (e.g., Grèzes et al., 2003; Filimon et al., 2007; Gazzola et al., 2007; Gazzola and Keysers, 2009; Caspers et al., 2010; Molenberghs et al., 2012; Kemmerer, 2021), which is thought to mediate AO effects. We used brain modeling software (see HD-tDCS section) to determine the tDCS montage for maximal focal stimulation of these ROIs. Therefore, we assumed that adding active HD-tDCS to AO would improve motor performance compared to AO only (with sham HD-tDCS). It appears that the addition of the current configuration of active HD-tDCS to the simultaneous AO interfered with the effects of observational learning (represented by the performance of the AO with sham HD-tDCS group). Interestingly, Enticott et al. (2012), who used large pad electrodes and a higher stimulation intensity (2 mA) than in the current study (1 mA), indeed found that active tDCS with an anodal or cathodal electrode over the IPL decreased the cortico-spinal excitability. It should be noted that inconsistent findings were found with respect to the effects of AO combined with tDCS (Wade and Hammond, 2015; Apšvalka et al., 2018). tDCS with an anodal electrode placed over the premotor cortex combined with AO improved serial reaction task performance (Wade and Hammond, 2015). However, conventional tDCS with an anodal electrode placed over M1 during AO did not significantly affect serial time performance (Apšvalka et al., 2018). The inconsistent findings may be related to the different ROIs that were targeted, the different parameters of stimulation (such as intensity and duration) and the use of conventional large pad tDCS in the above-mentioned studies.

The different motor responses to AO with and without active HD-tDCS in the current study are in line with previous evidence that AO effects on motor performance can be modified by different factors, for example the observed model type (Rohbanfard and Proteau, 2011), visual guidance during observation (D’Innocenzo et al., 2016), instruction type (Frenkel-Toledo et al., 2020), and observation viewpoint (Watanabe and Higuchi, 2016). Here, it seems that changes in brain excitability due to HD-tDCS over the IFG and IPL affected the response to AO. Measuring MEPs, using Transcranial Magnetic Stimulation, from UL muscles at rest before and after AO with and without tDCS (targeting the IPL and IFG) in future studies can elucidate this issue.

It should be noted, however, that Delta, but not Reaching Time, improved in the AO + active HD-tDCS group compared to the NM + sham HD-tDCS group in the posttest. The Delta reflects the difference between the response time of the unexpected and expected RMs toward unit 5. It is possible that the unexpected element of the Delta measure increased the difficulty of the task, therefore increasing the amount of room for improvement in Delta among healthy participants and the sensitivity to detect a change between the AO + active HD-tDCS and NM + sham HD-tDCS groups.

Several explanations can be proposed for the findings that AO combined with active HD-tDCS did not improve Reaching Time and Delta compared to AO alone and did not improve Reaching Time compared to observation of NM. First, different tDCS protocols, depending on several parameters such as stimulation intensity and duration, can even invert neurophysiological (Nitsche and Paulus, 2000; Batsikadze et al., 2013; Moliadze et al., 2015; Ammann et al., 2016; Strube et al., 2016) and behavioral effects (Ehrhardt et al., 2021; Lerner et al., 2021). Recent evidence suggests that tDCS with an anodal electrode over the ROI does not necessarily have an excitability effect, and vice versa with regard to tDCS with a cathodal electrode over the ROI (Batsikadze et al., 2013; Shilo and Lavidor, 2019; Lerner et al., 2021). For example, Batsikadze et al. (2013) found that conventional tDCS, with a cathodal electrode placed over M1 at 2 mA intensity for 20 min, led to increased MEP. Lerner et al. (2021) found that following 20 min of 2 mA HD-tDCS with an anodal electrode placed over M1, improvement in movement time of reaching performance from the course of stimulation to posttest was significantly lower compared to sham HD-tDCS. Shilo and Lavidor (2019), using a reaction time task, found that stimulation with a cathodal electrode placed over M1 at 2 mA led to faster performance than stimulation with a corresponding anodal electrode placed over M1 after 13 min of stimulation. It has been suggested that the change in cortical excitability from excitation to inhibition is related to neuronal inhibitory mechanisms that have a delayed onset when exposed to excitatory protocols (Parkin et al., 2019). Alternatively, neurons throughout the cortex may not be modulated in a homogenous manner. Neurons in deep cortical layers were often deactivated by anodal stimulation and activated by cathodal stimulation, contrary to superficially located cells (Purpura and McMurtry, 1965). Second, it is possible that in the AO + active HD-tDCS group, HD-tDCS activated inhibitory mirror neurons, which resulted in reduced AO-related motor improvement. Indeed, a subset of mirror neurons demonstrated excitation or inhibition during both action execution and AO, and a subset of these neurons demonstrated excitation during action execution and inhibition during AO (Mukamel et al., 2010). The latter neural subset of neurons may help preserve the sense of being the owner of an action during execution and exert control on unwanted imitation during AO. Indeed, participants were instructed to avoid moving during AO. It should be noted, however, that the activity of these neurons that demonstrated excitation during action execution and inhibition during AO was recorded from cells in human medial frontal and temporal cortices (Mukamel et al., 2010) and not specifically IPL and IFG. Furthermore, in the current study, HD-tDCS over the right IFG may have induced response inhibition. Activated response inhibition was suggested to relate to the right IFG based on studies which investigated the neural basis of inhibitory control (Aron et al., 2003, 2014; Bartoli et al., 2018; Hannah et al., 2020). Lesions to the right IFG were found to be associated with an elongation of stop-signal reaction time (Aron et al., 2003). According to a theory of inhibitory control, right IFG implements a brake over response tendencies (Aron et al., 2014).

### 4.1. Limitations

The study has several limitations. First, despite we used HD-tDCS and brain modeling software to determine the tDCS montage for maximal focal stimulation of the IPL and the IFG to improve spatial focality of current, stimulation was not exclusively delivered to IPL and IFG. The electric fields (V/m) could have differed between groups because of differences in the participants’ anatomical features (Laakso et al., 2015). Second, the current study design was a single-blind randomized controlled study because the experimenter was not blinded to group allocation. It should be noted, however, that the scoring of the motor task was automatically computed by the LabVIEW software. Third, as there is no consensus about the optimized tDCS protocol, it could be that using other stimulation parameters, such as stimulation intensity or duration, could have yielded different results. Fourth, the hMNS activity is enhanced *via* the observation of a movement (Rizzolatti and Craighero, 2004; Rizzolatti and Sinigaglia, 2016). However, additional control group which would have observed the same sequences of the lighted units but without reaching movement sequences toward these units would have verified whether improvement in motor performance was related specifically to the observed reaching movement sequences or the observed sequences of the lighted units.

## 5. Conclusion

Our results indicate that AO combined with the current montage of active HD-tDCS with maximal focal stimulation of the IPL and IFG, the main aggregates of the hMNS, interferes with the effects of AO alone on the motor performance of reaching movements. Future studies should consider applying MEP measurements to elucidate the electrophysiological changes in cortical excitation underlying behavioral effects.

## Data availability statement

The raw data supporting the conclusions of this article will be made available by the authors, without undue reservation.

## Ethics statement

The studies involving human participants were reviewed and approved by Ariel University Institutional Ethical Board (approval number: AU-HEA-SFT-20210721). The patients/participants provided their written informed consent to participate in this study.

## Author contributions

GS and SF-T conceived the study, analyzed the data, performed the statistical analysis, and drafted the manuscript. GS, ZK, DT, ME, and SF-T designed the study and interpreted the results. GS and DT acquired the data. All authors critically reviewed and edited the manuscript, read, and approved the final manuscript.

## Supplementary material

Supplementary Figure 1 Distribution of reaching time of each group displayed by time. ms, millisecond; AO, action observation; HD-tDCS, high-definition transcranial stimulation. Plus represents average. Asterisks denote a significant difference.

Supplementary Figure 2 Distribution of delta of each group displayed by time. ms, milliseconds; AO, action observation; HD-tDCS, high-definition transcranial stimulation. Plus represents average. Asterisks denote a significant difference.
