# Supplementary material for: The effect of action observation combined with high-definition transcranial direct current stimulation on motor performance in healthy adults: A randomized controlled trial
Source: Front Hum Neurosci. 2023 Mar 1;17:1126510. doi: 10.3389/fnhum.2023.1126510 (PMC10014919; doi:10.3389/fnhum.2023.1126510)
Supplement: Supplementary Figure 1 — Distribution of reaching time of each group displayed by time. ms, millisecond; AO, action observation; HD-tDCS, high-definition transcranial stimulation. Plus represents average. Asterisks denote a significant difference. [file Data_Sheet_1.pdf]

Supplementary Figure 1

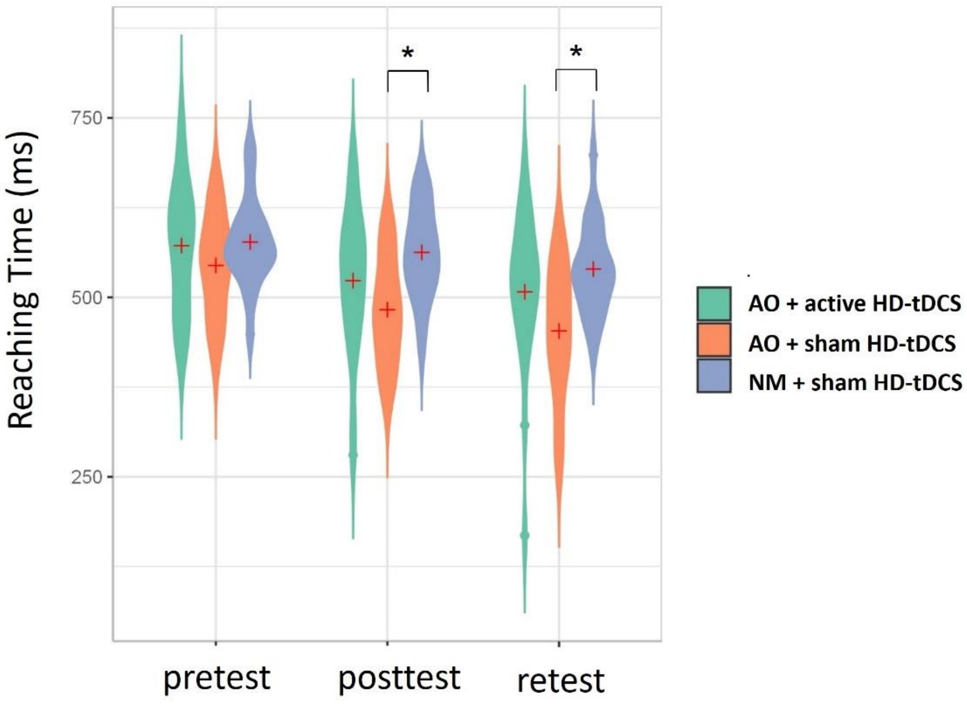

Title: Distribution of Reaching Time of each group displayed by time.

Legend: ms = millisecond; AO = action observation; tDCS = transcranial direct current stimulation. Plus represents average. Asterisks denote a significant difference.

Supplementary Figure 2

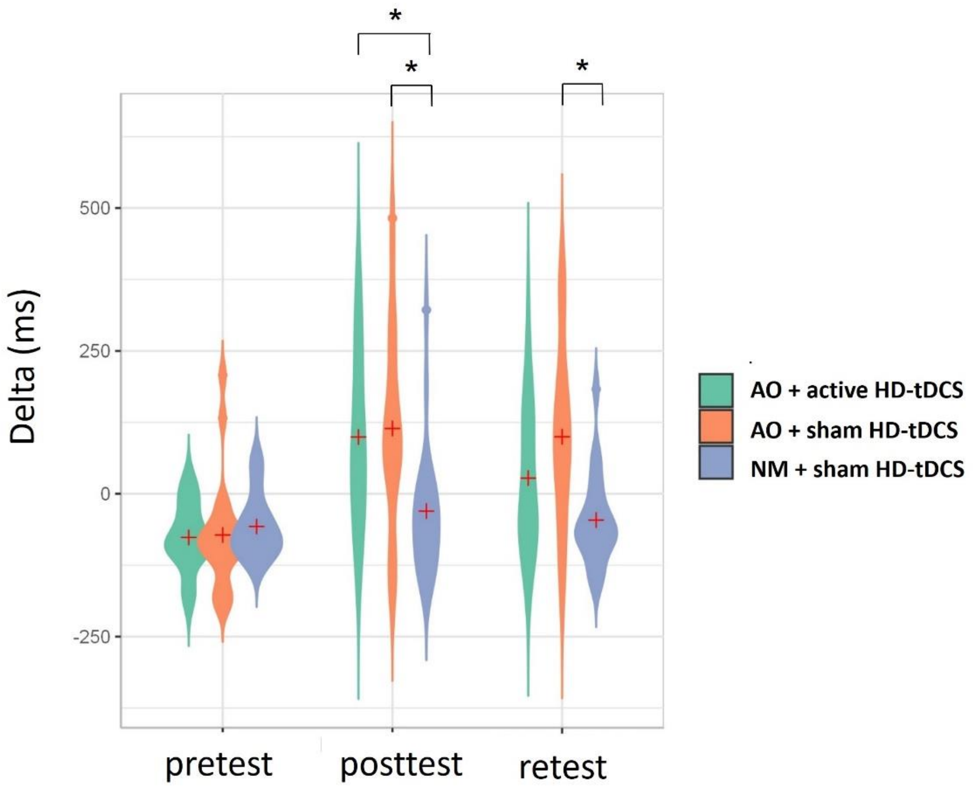

Title: Distribution of Delta of each test displayed by groups.

Legend: ms = milliseconds; AO = action observation; tDCS = transcranial direct current stimulation. Asterisks denote a significant difference.
